# Biophysical and functional characterization of the human TAS1R2 sweet taste receptor overexpressed in a HEK293S inducible cell line

**DOI:** 10.1038/s41598-021-01731-3

**Published:** 2021-11-15

**Authors:** Christine Belloir, Marine Brulé, Lucie Tornier, Fabrice Neiers, Loïc Briand

**Affiliations:** Centre des Sciences du Goût et de l’Alimentation, AgroSup Dijon, CNRS, INRAE, Université Bourgogne Franche-Comté, 21000 Dijon, France

**Keywords:** Biochemistry, G protein-coupled receptors, Membrane proteins

## Abstract

Sweet taste perception is mediated by a heterodimeric receptor formed by the assembly of the TAS1R2 and TAS1R3 subunits. TAS1R2 and TAS1R3 are class C G-protein-coupled receptors whose members share a common topology, including a large extracellular N-terminal domain (NTD) linked to a seven transmembrane domain (TMD) by a cysteine-rich domain. TAS1R2-NTD contains the primary binding site for sweet compounds, including natural sugars and high-potency sweeteners, whereas the TAS1R2-TMD has been shown to bind a limited number of sweet tasting compounds. To understand the molecular mechanisms governing receptor–ligand interactions, we overexpressed the human TAS1R2 (hTAS1R2) in a stable tetracycline-inducible HEK293S cell line and purified the detergent-solubilized receptor. Circular dichroism spectroscopic studies revealed that hTAS1R2 was properly folded with evidence of secondary structures. Using size exclusion chromatography coupled to light scattering, we found that the hTAS1R2 subunit is a dimer. Ligand binding properties were quantified by intrinsic tryptophan fluorescence. Due to technical limitations, natural sugars have not been tested. However, we showed that hTAS1R2 is capable of binding high potency sweeteners with *K*_d_ values that are in agreement with physiological detection. This study offers a new experimental strategy to identify new sweeteners or taste modulators that act on the hTAS1R2 and is a prerequisite for structural query and biophysical studies.

## Introduction

Taste detection is mediated by a single heteromeric receptor composed of two G-protein coupled receptors (GPCRs), named TAS1R2 (taste receptor type 1, member 2) and TAS1R3 (taste receptor type 1, member 3)^1–6^. TAS1R2 and TAS1R3 subunits are members of the class C GPCR family that includes the metabotropic glutamate receptors (mGluRs), the calcium-sensing receptor (CaSR), and the γ-aminobutyric acid receptor B (GABA_B_R)^7^. GPCR class C architecture is composed of a large N-terminal domain (NTD) composed of the Venus flytrap (VFT) module linked to the characteristic heptahelical transmembrane domain (TMD) by a short cysteine rich domain (CRD). The TAS1R2/TAS1R3 sweet taste receptor is able to detect a wide variety of sweet tasting compounds, including natural sugars, sugar alcohols, and artificial and natural sweeteners^8,9^. Mouse-human chimaera, site-directed mutagenesis studies and molecular modelling have revealed that the VFT module of TAS1R2 contains the primary binding site for sweet tasting compounds, where natural sugars (sucrose, glucose and fructose) and non-caloric sweeteners (aspartame, neotame, sucralose, saccharin-Na, rebaudioside and acesulfame-K) bind^3,10–16^. Molecular modelling experiments have revealed that binding of sweeteners to the VFT of TAS1R2 leads to major conformational changes to the TMDs of TAS1R2 and TAS1R3, leading to G protein activation^17^. However, at least three other ligand-binding sites have been identified on the TAS1R2/TAS1R3 sweet taste receptor. One binding site is located in the TAS1R3-VFT module, where natural sugars (sucrose, fructose and glucose) and the chlorodeoxysugar sucralose have been found to bind^13,18^. Another binding site is located in TAS1R3-TMD, where the sweeteners cyclamate and neohesperidin dihydrochalcone and the sweet taste inhibitors lactisole and gymnemic acid bind^19–23^. Although the functional role of the CRD of TAS1R3 remains to be elucidated, it has been shown that this domain is also involved in the response to sweet tasting proteins, including brazzein and thaumatin^24,25^. Additionally, it has been shown that TAS1R2-TMD interacts with the sweeteners S-819 and perillartine^26,27^ and with the sweet taste modulator amiloride^28^. The presence of multiple binding sites on the sweet taste receptor explains the synergy observed between some sweetener mixtures^9,27,29^.

Ligand binding studies conducted with the mouse and human TAS1R2- and TAS1R3-VFTs expressed in *Escherichia coli* have shown that both these proteins are able to bind natural sugars and the chlorodeoxysugar sucralose with distinct and physiologically relevant affinities^13,18^. In addition, Nie and collaborators revealed that mouse TAS1R3-VFT binds sucrose with a higher affinity than TAS1R2-VFT, though this relationship is reversed for glucose^13^. More recently, human TAS1R2-VFT has been expressed in *E. coli* as a fusion protein with the small ubiquitin-like modifier (SUMO). Using NMR and various biophysical approaches, TAS1R2-VFT was shown to bind the dipeptide sweetener neotame with a dissociation constant (*K*_d_) value in the micromolar range^30^. An interesting approach was also recently developed allowing a large-scale co-expression of TAS1R extracellular domains from Medaka fish (mf). The two subunits mfTAS1R2-VFT/mfTAS1R3-VFT were expressed and purified in a heterodimeric state using Drosophila S2 cells^31^. The high expression of both subunits enabled biophysical and structural analyses such as isothermal calorimetry and small-angle X-ray scattering for the detection of ligand binding and conformational change upon taste substance binding^32^, and structure resolution by crystallization^14^. mfTAS1R2-VFT/mfTAS1R3-VFT heterodimer responded to a wide variety of l-amino acids (l-alanine, l-glutamine), but not to sugars or other sweeteners^33^.

The relative contribution of the two subunits constituting the heterodimeric human TAS1R2/TAS1R3 receptor remains largely unknown. This lack of knowledge is partly due to difficulties in preparing sufficient quantities of functional VFT domains suitable for ligand binding assays and biophysical analysis. We previously reported that hTAS1R3-VFT and cat TAS1R1-VFT could be expressed as functional proteins in *Escherichia coli*^18,34,35^. The VFT domain was expressed in inclusion bodies with a high yield as previously reported^18,34,35^. To further understand the structural basis of sweet compound recognition by the sweet taste receptor, we produced the full-length human TAS1R2 (hTAS1R2) protein. For this purpose, hTAS1R2 was overexpressed using a stable tetracycline-inducible HEK293S cell line^36–39^. The subunit was purified after detergent solubilization, and its correct fold was confirmed using circular dichroism (CD). Using intrinsic tryptophan fluorescence, we demonstrated that hTAS1R2 is able to bind the four sweeteners sucralose, neotame, acesulfame-K and perillartine with physiologically relevant affinities. To demonstrate that the binding is specific, we tested the sweetener cyclamate that binds to hTAS1R3-TMD. This study provides new insights into the molecular determinants of sweet taste perception and opens ways to screen new sweet tasting compounds or taste modulators.

## Results

### Expression of hTAS1R2 in the HEK293S GnTI- inducible cell line

**Figure 1:**
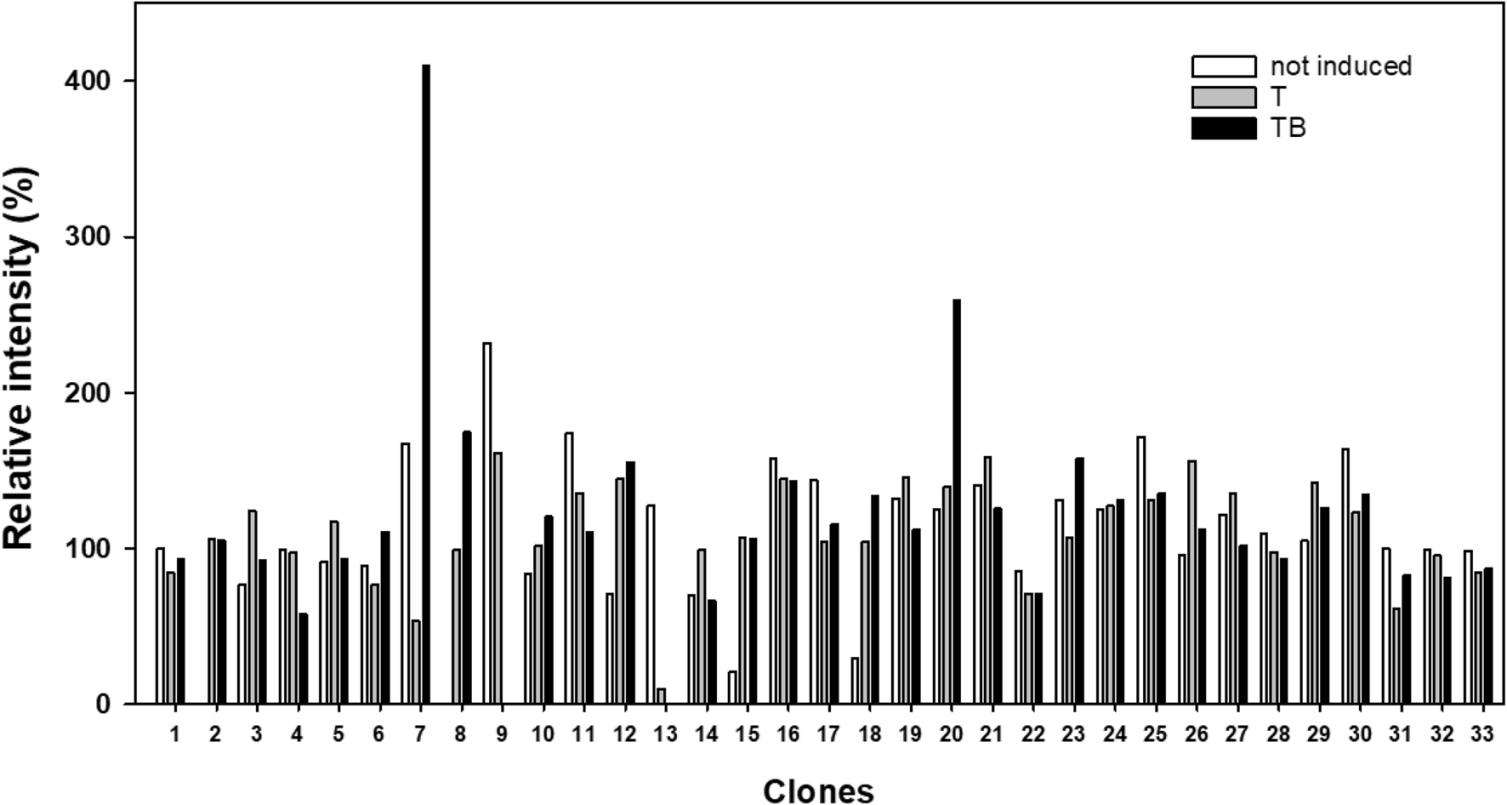
Immunoblot analysis of HEK293S-GnTI- clones stably transfected with pcDNA5/TO-FLAG-hTAS1R2. Each clone was tested for induction with tetracycline alone (1 µg/mL) or in combination with 5 mM NaBu. Three microliters of solubilized protein from cell lysates (3 µg) were loaded onto PVDF membranes, analysed using the dot blot technique and probed with monoclonal FLAG antibody. The results were quantified using Image Lab (Bio-Rad) and normalized to 100% relative intensity. T: tetracycline induction; TB: tetracycline and NaBu induction. Dot blot image is presented in Supplementary Fig. S1.

**Figure 2:**
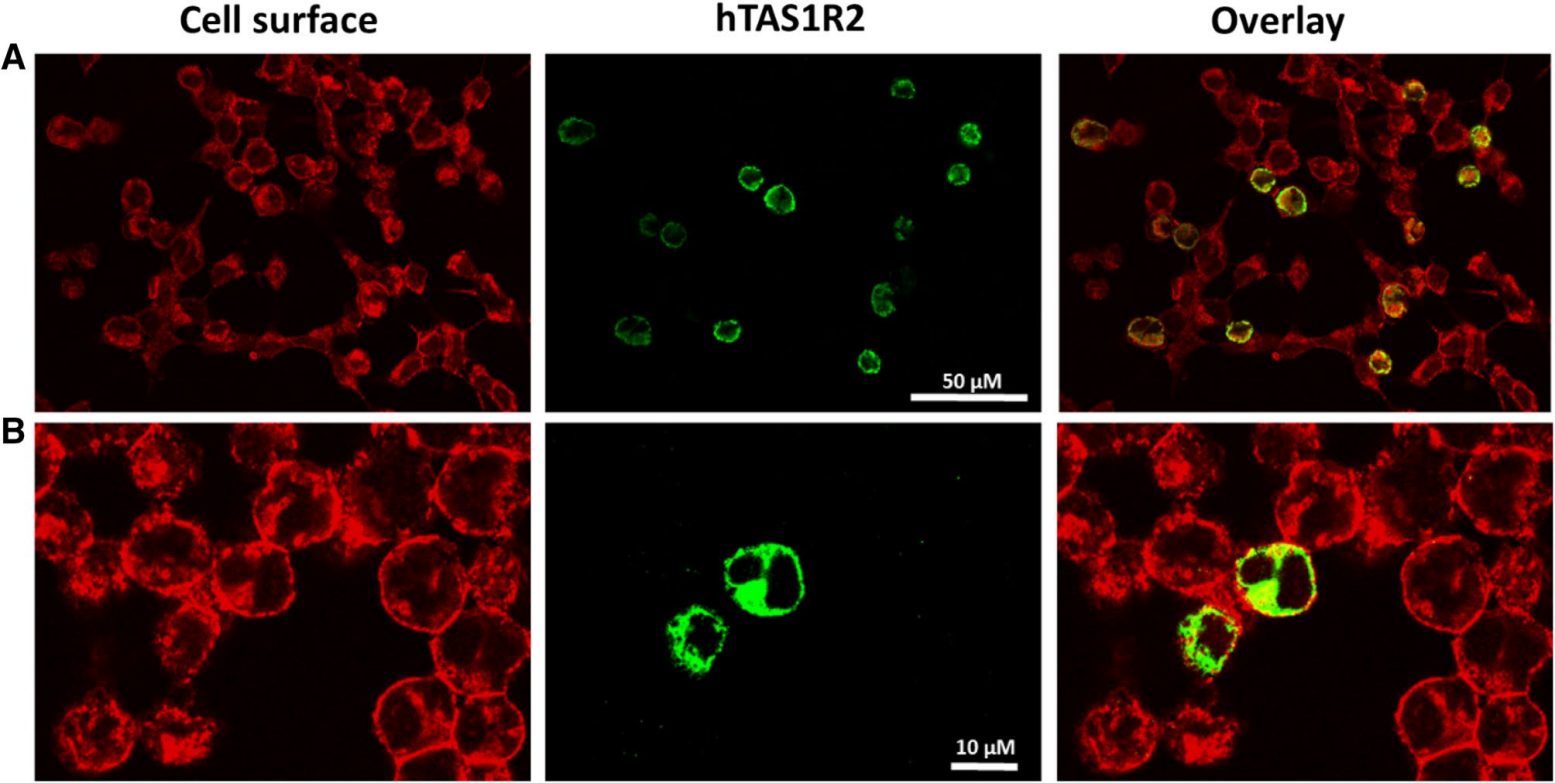
Immunocytochemistry of hTAS1R2-inducible HEK293S cells treated with tetracycline and NaBu. Cells from clone 7 were treated with 1 µg/mL tetracycline and 5 mM NaBu for 48 h. The level of induced hTAS1R2 protein (shown in green) was detected using a primary anti-FLAG M2 antibody and a fluorescently labelled secondary antibody (Alexa Fluor 488). The cell surface (shown in red) was detected by biotin-conjugated concanavalin A and streptavidin-conjugated Alexa Fluor 568. The overlay images indicate the localization of the receptor at the cell surface (shown in yellow). (**A**) The cells were analysed using an epi-fluorescence inverted microscope (Eclipse TiE, Nikon, Champigny sur Marne, France) equipped with an ×20 objective lens and a LucaR EMCCD camera (Andor Technology, Belfast, UK). (**B**) The cells were analysed using a two photon confocal microscope (Nikon A1-MP) equipped with an ×60 objective lens (DImaCell platform, University of Burgundy, Dijon, France).

After transfection with the pcDNA5/TO-FLAG-hTAS1R2 plasmid and antibiotic selection, thirty-three HEK293S-GnTI- clones were subjected to induction in media supplemented with tetracycline or a combination of tetracycline and NaBu. To detect hTAS1R2 protein, we used the highly sensitive and specific anti-FLAG M2 tag antibody. Dot blot analysis of the cell lysates revealed that tetracycline alone does not allow an induction of the selected clones. In contrast, for two clones, the combination of tetracycline and NaBu allowed the induction of high amounts of FLAG-tagged hTAS1R2. The highest expression was observed with clone 7 (Fig. 1, Supplementary Fig. S1). Consequently, this clone was selected for subsequent experiments. Cell-based immunocytochemistry experiments confirmed expression of hTAS1R2 using a combination of tetracycline and NaBu (Fig. 2). The subcellular localization of the hTAS1R2 protein was investigated using confocal microscopy. This analysis revealed that most of the expressed protein is localized intracellularly with a minor portion present at the cell surface.

### Detergent screening and purification of hTAS1R2

**Figure 3:**
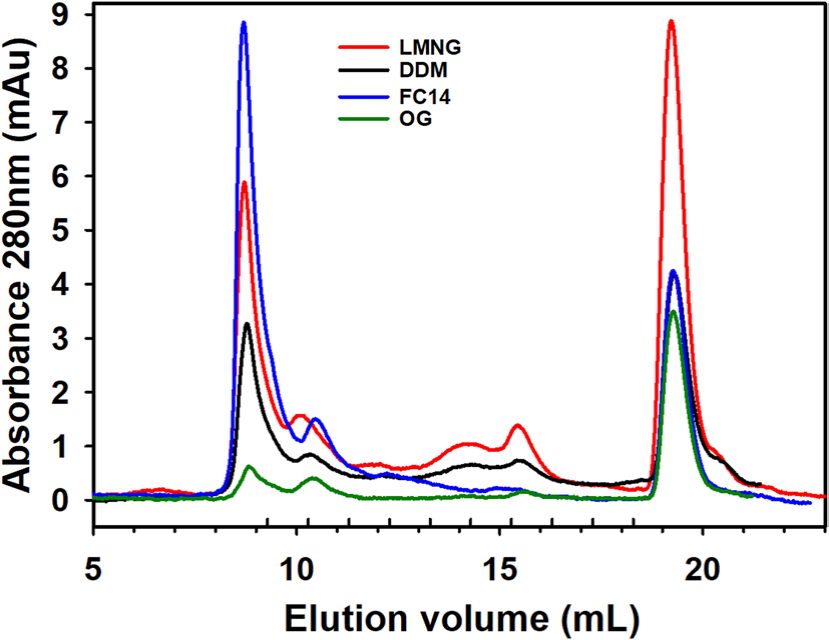
Detergent screen for optimal solubilization and purification of hTAS1R2 expressed in HEK293S cells. Expression of hTAS1R2 was induced with tetracycline (1 µg/mL) and NaBu (5 mM) for 48 h. The hTAS1R2 receptor was solubilized in PBS containing detergent at a concentration of 2% (w/v) for 2 h at 4 °C. After FLAG immunoaffinity purification, the eluate was concentrated to 0.3 mg/mL, loaded on a Superdex 200 3.2/300 column and eluted with PBS containing 0.1% detergent (pH 7.3) at 0.5 mL/min. Purified hTAS1R2 eluted at approximately 13.7 mL. Detergent abbreviations: LMNG, Lauryl maltose neopentyl glycol; DDM, n-Dodecyl-β-d-maltopyranoside; FC14, Fos-choline 14; OG, Octyl-β-d-glucoside.

To purify the expressed hTAS1R2, we first investigated which detergent was able to solubilize hTAS1R2. Consequently, we performed a small detergent screen that included the main detergents that have been successfully used to solubilize several other GPCRs^40–44^. These detergents include zwitterionic fos-choline 14 (FC14) and three non-ionic detergents, dodecyl maltoside (DDM), octyl glucoside (OG) and lauryl maltose neopentyl glycol (LMNG). The hTAS1R2 solubilized with the different detergents was purified using FLAG immunoaffinity and analysed using gel filtration (Fig. 3). We found that the purified hTAS1R2 eluted at approximately 13.7 mL, corresponding to a molecular weight of 107 kDa, as calculated from the gel filtration calibration curve (Supplementary Fig. S2). The chromatogram revealed an increase in absorbance between 13.5 and 15 mL for LMNG, DDM and, to a lesser extent, for FC14. Functional evaluation of these purified fractions using intrinsic fluorescence revealed that only hTAS1R2 extracted by LMNG and FC14 was able to bind sucralose, whereas hTAS1R2 extracted with DDM and OG led to a non-functional receptor. Since LMNG allowed us to obtain higher amount of functional receptor, this detergent was selected for all subsequent analyses.

**Figure 4:**
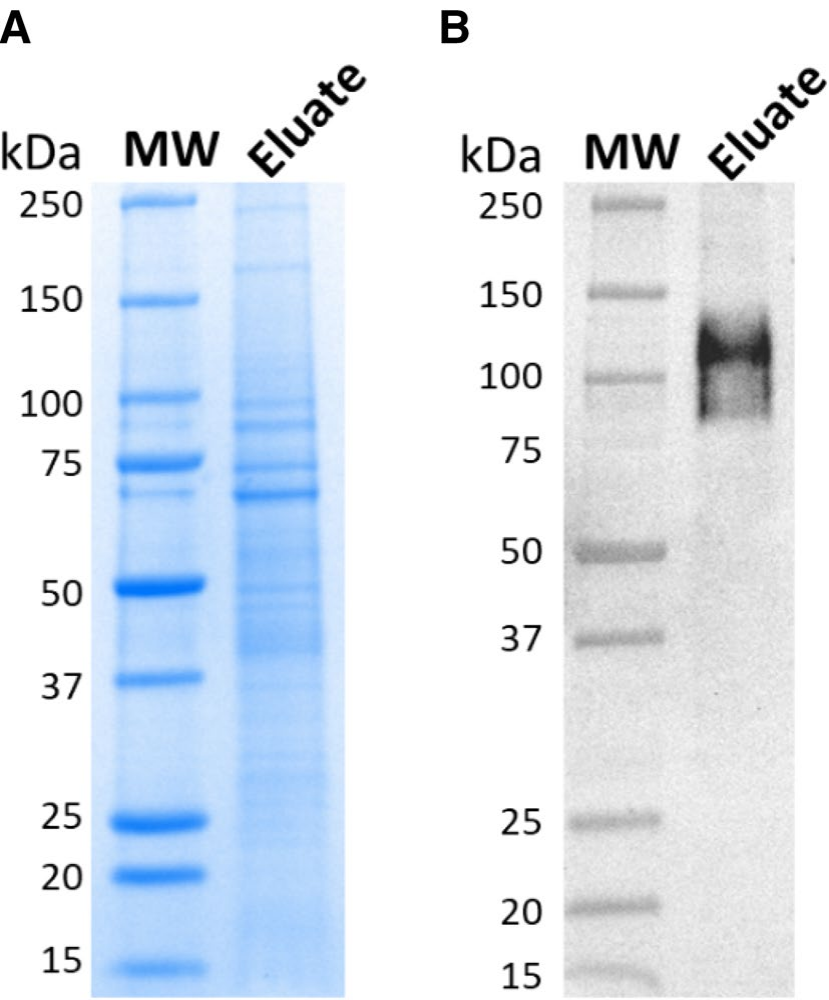
Analysis of immunoaffinity purified hTAS1R2. FLAG-tagged hTAS1R2 was solubilized in PBS containing 2% LMNG and captured using the EZview Red anti-FLAG M2 affinity gel. After elution with FLAG peptide, the eluate was collected, concentrated and subjected to SDS-PAGE followed by (**A**) staining with Coomassie blue and (**B**) by western blotting using mouse anti-FLAG M2 primary antibody and goat anti-mouse horseradish peroxidase conjugated secondary antibody. Full-length gels/blots are presented in Supplementary Fig. S3.

The LMNG-solubilized hTAS1R2 receptor was purified using a two-step purification process. Coomassie blue SDS-PAGE analysis combined with western blot analysis of the protein purified by anti-FLAG M2 affinity chromatography showed four main bands migrating between 70 and 100 kDa and two other, less intense, migrating bands at 167 and 208 kDa (Fig. 4A, Supplementary Fig. S3A). Western blot analysis confirmed the presence of FLAG-tagged hTAS1R2 protein migrating around the expected molecular weight (115 kDa) (Fig. 4B, Supplementary Fig. S3B).

**Figure 5:**
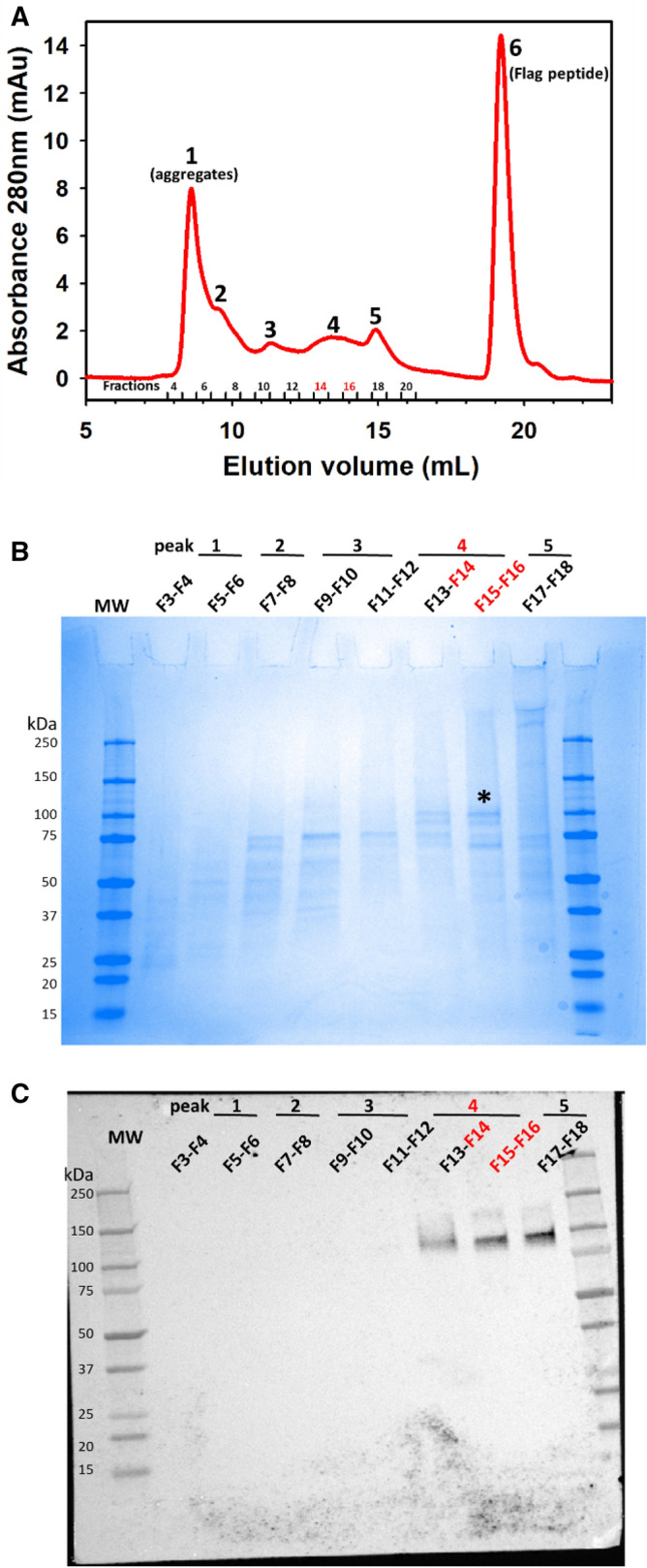
Size exclusion chromatography of immunoaffinity-purified hTAS1R2. (**A**) SEC analysis was performed on an Akta Pure FPLC system equipped with a Superdex 200 Increase 10/300GL column (GE Healthcare). Immunoaffinity-purified hTAS1R2 was eluted using PBS-LMNG 0.1% (pH 7.3). Six distinct peaks were observed, suggesting the presence of several oligomeric forms of the protein. The peak fractions were analysed by (**B**) SDS-PAGE followed by staining with Coomassie blue and (**C**) by western blotting using mouse anti-FLAG M2 primary antibody and goat anti-mouse horseradish peroxidase conjugated secondary antibody. The peak and fraction numbers refer to those designated in (**A**). Peak 4 (fractions 14–16) contained mainly hTAS1R2 marked with a black asterisk.

To further purify hTAS1R2 and remove the FLAG peptide used for protein elution, the immunoaffinity-purified FLAG-tagged hTAS1R2 protein was subjected to gel filtration analysis. Six peaks were observed (Fig. 5A). Peaks 1 and 6 corresponded to aggregates and the FLAG peptide, respectively. SDS-PAGE and western blot analysis using anti-FLAG M2 antibody showed that peaks 4 and 5 mainly contained monomeric hTAS1R2 that migrated at 100 kDa. However, three other bands migrating at 92, 76 and 71 kDa were detected by Coomassie blue staining. Peaks 2 and 3 showed an intense band migrating at approximately 75 kDa, as observed by SDS-PAGE but not by western blotting (Fig. 5B,C). The total yield of the hTAS1R2 protein from peak 4 (fractions 14–16) resulting from sixty T225 flasks was approximately 135 µg (2.2 µg/flask, i.e., 0.08–0.1 µg/10^6^ cells in terms of cell productivity).

### Secondary structure and oligomerization analysis of the purified hTAS1R2

**Figure 6:**
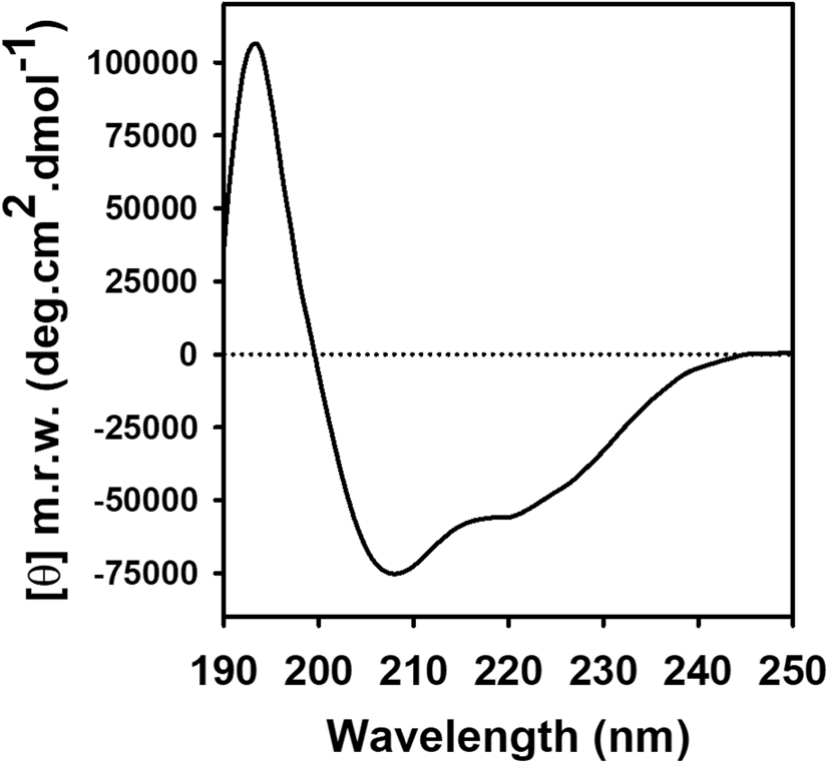
Secondary structure analysis of purified hTAS1R2 using circular dichroism spectroscopy. The far UV CD spectrum of hTAS1R2 recorded in PBS at 0.1% LMNG pH 7.3 shows the presence of a high content of α-helical secondary structures. Protein concentration 1 mg/mL. Light path 0.01 cm.

Circular dichroism (CD) spectroscopy was used to confirm the structural integrity of hTAS1R2 purified by gel filtration (peak 4). The far-UV spectrum of purified hTAS1R2 displayed a positive peak centred at 193 nm and two negative peaks at 208 and 222 nm (Fig. 6), which clearly showed the helical character of the protein. Deconvolution of the CD spectrum revealed that hTAS1R2 was approximately 66% α-helix and 18% β-sheet. This composition is consistent with the secondary structure content of other crystallized class C GPCRs, such as mGluR (PDB 6N52)^45^ and GABAb (PDB 4MS4)^46^ for which the protein circular dichroism data bank (PCDDB) calculated around 37% α-helix for both proteins, and 12% and 21% β-sheet, respectively^47^.

**Figure 7:**
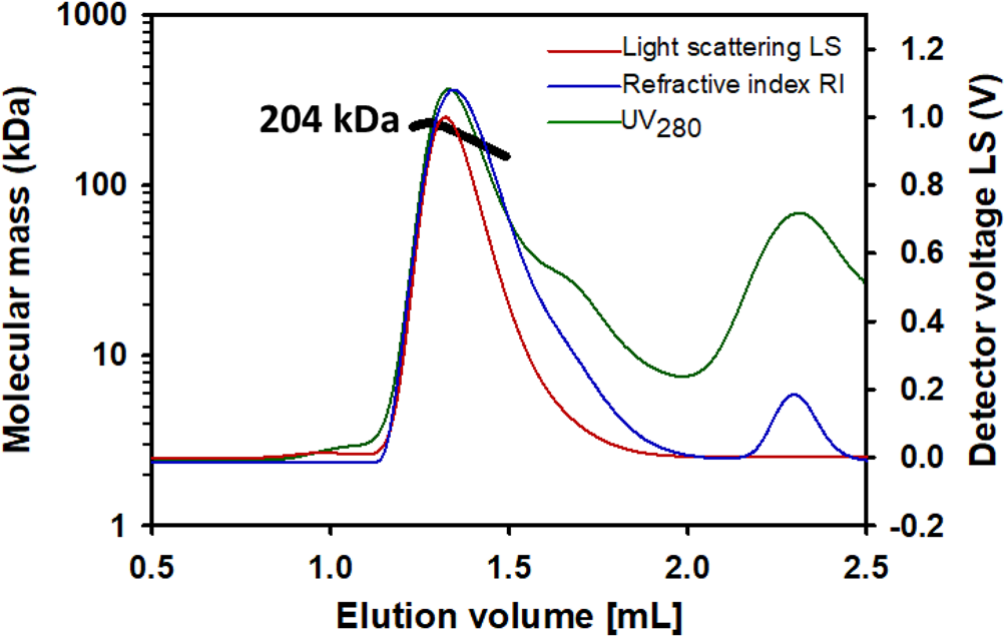
Oligomeric state analysis of purified hTAS1R2. After size exclusion chromatography (SEC) and separation with a Superdex 200 3.2/300 column (GE Healthcare), the molecular mass was determined from the Raleigh ratio, measured by static light scattering and the refractive index. The calculated molecular mass (bold black curve), refraction index (blue curve), light scattering (red curve) and UV at 280 nm (green curve) are shown. A main oligomeric form with a measured mass of 204 kDa was detected, indicating the presence of a dimeric hTAS1R2 form associated with detergents.

To confirm the oligomerization state of FLAG-tagged hTAS1R2, fractions 14 to 16 corresponding to peak 4 were pooled and analysed using an online size exclusion chromatography (SEC) coupled to MALS, RI and UV detectors. SEC-MALS allows determination of the direct molecular mass of protein detergent complexes and does not require calibration curves^48^. Nevertheless, because the protein was obtained in LMNG detergent at a concentration in buffer above the critical micelle concentration, the quality of the chromatographic data was validated using two known molecular markers, bovine serum albumin (BSA) and β-amylase (Supplementary Fig. S4). Thus, the detergent complexes containing a BSA monomer or β-amylase were correctly resolved, with calculated molecular weights of 66 kDa and 200 kDa, respectively (theoretical molecular weights of 66 kDa and 200 kDa, respectively). The UV analysis at 280 nm-LS (90° angle)-RI overlay of the purified hTAS1R2 revealed the oligomeric states and the presence of the receptor in heterogeneous forms (Fig. 7). A predominant dimeric form with a measured mass of 204 kDa was detected after injection of concentrated fractions resulting from gel filtration. The SEC-MALS analysis of the linear and cumulative distribution of the molar mass confirmed that the dimers represented 80% of the total amount, while 20% was still present in monomeric form with an average molecular weight of 100 kDa. The theoretical mass of FLAG-tagged hTAS1R2 is 96.2 kDa; thus, hTAS1R2 appeared mainly as a dimer associated with detergents.

### Ligand binding properties of the purified hTAS1R2

**Figure 8:**
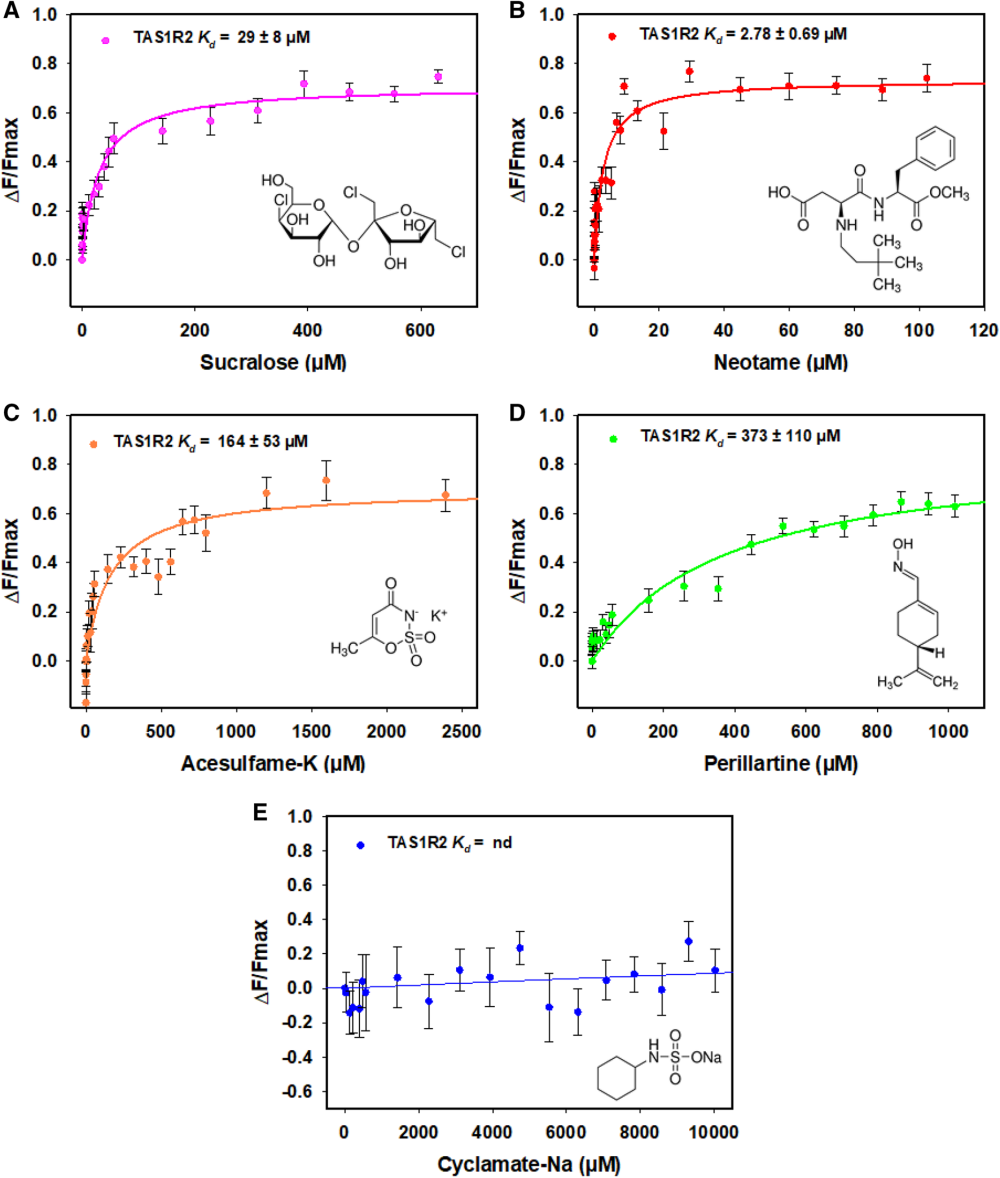
Binding activity of the purified hTAS1R2 using intrinsic tryptophan fluorescence. Intrinsic fluorescence was measured using a Cary Eclipse spectrofluorimeter. Dose–response relationship of hTAS1R2 fluorescence (λex = 280 nm, λem = 340 nm) was observed following sweetener addition. The data were fitted with a standard nonlinear regression method using SigmaPlot software. Data represent mean ± sem from at least four independent experiments. The reported *K*_d_ values are the average of triplicate measurements from at least three independently purified protein samples.

To characterize the interactions of purified hTAS1R2 with its ligands, we determined the dose–response relationship of its intrinsic tryptophan fluorescence upon titration with sweeteners previously demonstrated to bind the hTAS1R2 subunit^3,10–12,15,16,26,28^. We first tested the ability of neotame, sucralose, and acesulfame-K to bind to hTAS1R2. These compounds have been shown to bind hTAS1R2-VFT^10,12,15,16^. We found that the addition of neotame, sucralose, and acesulfame-K increased the fluorescence intensity of hTAS1R2. We observed that neotame was the highest affinity ligand, exhibiting a *K*_d_ value of 2.78 ± 0.69 µM (Fig. 8B) whereas sucralose and acesulfame-K bound hTAS1R2 with lower affinities (*K*_d_ values of 29 ± 8 µM and 164 ± 53 µM, respectively) in agreement with their lower potencies (Fig. 8A,C,D). We also tested the sweetener perillartine shown to activate the monomeric hTAS1R2 receptor and bind its TMD^26–28^. We found that perillartine interacts with hTAS1R2 with lower affinity leading to a *K*_d_ value of 373 ± 110 µM (Fig. 8A,C,D). As a negative control, we tested the sweetener cyclamate, which has been shown to bind hTAS1R3-TMD^21,23^. As expected, cyclamate addition had no effect on the intrinsic fluorescence of hTAS1R2 (Fig. 8E). Altogether, these data demonstrated that purified hTAS1R2 protein is functional and able to specifically bind sweet tasting molecules with micromolar affinities.

**Figure 9:**
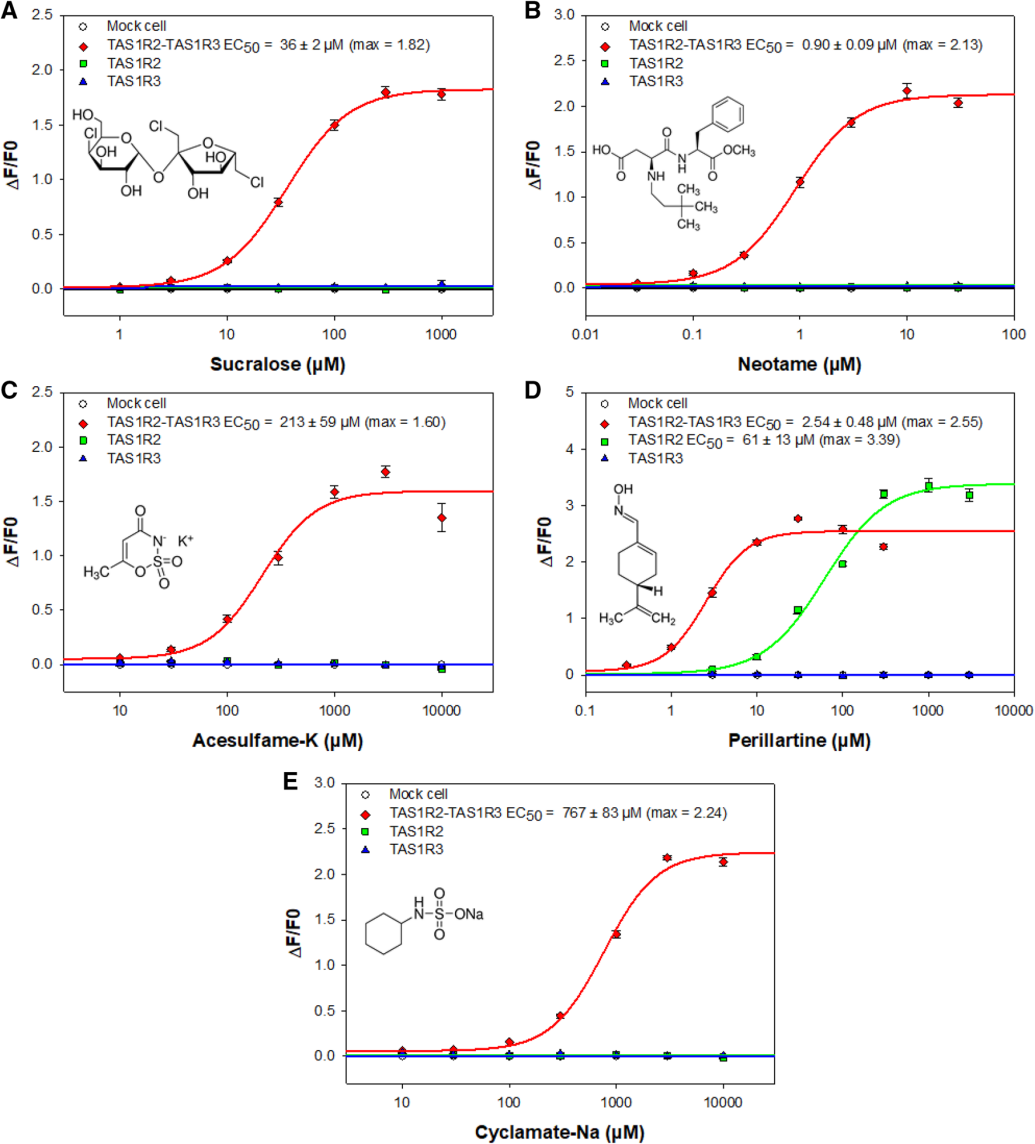
Human TAS1R2/TAS1R3 dose–response curves with different sweeteners. HEK293T-Gα16gust44 cells were transiently transfected with pGP-CMV-GCaMP6S (fluorescent calcium biosensor) combined with pcDNA6-hTAS1R2-FLAG and pcDNA4-hTAS1R3-FLAG (red line), or with pcDNA6-hTAS1R2-FLAG alone (green line) or pcDNA4-hTAS1R3-FLAG alone (blue line) or empty expression vector alone (mock cell) for the control (white circles, solid black line). Sweet taste stimuli were automatically applied to the transfected cells, and fluorescence changes were monitored using a FlexStation 3. The logarithmically scaled x-axis indicates the sweetener concentration in µM, and the y-axis shows the variation in fluorescence upon agonist application. Sucralose (**A**), neotame (**B**), acesulfame-K (**C**), perillartine (**D**), and cyclamate-Na (**E**). Data represent mean ± sem of eighteen wells from three independent experiments.

To confirm the ligand binding data obtained with the purified hTAS1R2, we performed cellular assays to determine the functional activity of the heterodimeric sweet taste receptor^49,50^. First, the transient transfection rate was evaluated by immunostaining and showed that around 45% of cells expressed hTAS1R2 and hTAS1R3 proteins (Supplementary Fig. S5). Then, HEK293T-Gα16gut44 cells were transiently co-transfected with hTAS1R2-FLAG, hTAS1R3-FLAG and pGP-CMV-GCaMP6S (fluorescent calcium indicator) and stimulated with sweeteners. The lowest EC_50_ value (Fig. 9B) was measured for neotame (0.90 ± 0.09 µM), whereas cyclamate had the highest EC_50_ value (767 ± 83 µM) (Fig. 9E). Sucralose and acesulfame-K activated human sweet taste receptor with intermediate EC_50_ values (36 ± 2 and 213 ± 59 µM, respectively) (Fig. 9A,C). These values are in accordance with those previously reported^12,26,51,52^ and are in agreement with sweetener potencies (Table 1). We also tested the sweetener perillartine, which has been demonstrated to bind to hTAS1R2-TMD^26,28^. In addition to activate the hTAS1R2/hTAS1R3 heterodimer (2.54 ± 0.48 µM), perillartine is capable of activating hTAS1R2 in the absence of hTAS1R3 (61 ± 13 µM)^26,28^. We found that the perillartine-induced dose–response was strongly shifted towards a higher concentration range for the hTAS1R2 subunit expressed alone, with a slight increase in signal amplitude (by approximately 1.3-fold), compared to the responses of the heterodimeric sweet taste receptor (Fig. 9D). Our data confirm that hTAS1R2 alone, probably acting as a homodimer, is functional.

**Table 1:** Biochemical, biological and physiological features of sweet tasting compounds.

	*K*_d_ (µM)	EC50 (µM)			Relative sweetness
	hTAS1R2	hTAS1R2/hTAS1R3	hTAS1R2	hTAS1R3	
Neotame	2.78 ± 0.69	0.90 ± 0.09	n.r.	n.r.	11,000
Perillartine	373 ± 110	2.54 ± 0.48	61 ± 13	n.r.	2000
Sucralose	29 ± 8	36 ± 2	n.r.	n.r.	600
Acesulfame-K	164 ± 53	213 ± 59	n.r.	n.r.	200
Cyclamate-Na	n.b.	767 ± 83	n.r.	n.r.	30–50

*K*_d_ values were determined by intrinsic tryptophan fluorescence. EC_50_ values were calculated from the ligand dose–response relationship in HEK293T-Gα16gust44 cells transiently transfected with plasmid encoding pGP-CMV-GCaMP6S (fluorescent calcium biosensor), hTAS1R2-FLAG and/or hTAS1R3-FLAG. The relative sweetness of each sweetener is described on a molar basis.

n.r. = no response; n.b. no binding.

## Discussion

In this study, the codon-optimized hTAS1R2 gene was overexpressed in the order of few hundreds of micrograms using the tetracycline-inducible HEK293S GnTI- cell line. Insertion of an N-terminal FLAG epitope tag allowed purification and detection of the recombinant membrane protein. We demonstrated the ability of the detergent LMNG to efficiently extract and solubilize hTAS1R2, maintaining its functional activity. LMNG is an emerging detergent that has been highlighted for its remarkable ability to enhance structural stability while protecting protein activity^53–56^. Recently, LMNG has allowed the successful crystallization of several delicate membrane proteins, such as the class A GPCR rhodopsin bound to arrestin^57^, and the class B GPCR calcitonin receptor coupled to its heterotrimeric Gs protein complex^58^. Associated with cholesterol hemisuccinate (CHS), LMNG has been used recently to determine the structure of the full-length mGluR5 by cryo-EM^45^. In our conditions, the addition of CHS during the extraction and solubilization step increased the amount of extracted protein but unfortunately led to a loss of functionality.

SEC-MALS was used to determine the molecular mass of the purified FLAG-tagged hTAS1R2 protein. This analysis revealed that recombinant hTAS1R2 is mainly present in its dimeric form. Previous studies on mGluR2 demonstrated that class C GPCR dimerization is required to induce agonist activation and G-protein coupling^17,59^. It has been shown that hTAS1R3 surface expression requires hTAS1R2 co-expression in a specific membrane trafficking system different from that of mice^60^. More recently, the structural architecture of the heterodimeric sweet taste receptor was explored and it was revealed that the TAS1Rs C-terminus of the CRD needs to be properly folded for TAS1R3 dimerization and co-trafficking, but not for surface expression of TAS1R2^61^. In this study, the cell surface expression of most FLAG-tagged TAS1R2 mutant libraries was very low, with most protein remaining inside the cell, in accordance with the low expression level we observed. The authors demonstrated that inhibition of surface expression of TAS1R2 is associated with an altered sequence at the C-terminus in the transmembrane domain or cytosolic tail of TAS1R2. The authors highlighted conserved surfaces on the ECD and TMD for dimerization with TAS1R3^61^. However, it is unclear whether the TAS1R2 homodimer structure is physiological or could represent an alternative conformational state, such as the mGluR2 homodimer or heterodimer (mGluR2-mGluR4)^62^. Nevertheless, a cellular assay showed that TAS1R2 transiently expressed alone in absence of TAS1R3 subunit is able to be activated by perillartine, a sweet tasting compound that interacts with TAS1R2-TMD^26,28,63^.

High concentrations of sugars (i.e. fructose, glucose, sucrose) may modify the buffer polarity affecting tryptophan environment leading to unspecific fluorescent changes. For this reason, we characterized the functional activity of the purified hTAS1R2 by measuring its binding affinity with high potency sweeteners that have been previously shown to activate the heterodimeric sweet taste receptors. For this purpose, we monitored the changes in the intrinsic tryptophan fluorescence of hTAS1R2 as it contains 15 tryptophan residues, 13 of which are localized in the NTD. Except for cyclamate, which is known to bind to TAS1R3-TMD, addition of all of the tested sweeteners led to an increase in fluorescence of the full-length hTAS1R2, which was saturable. Using this technique, we successfully measured *K*_d_ values, which were in the micromolar range. The measured *K*_d_ values for sucralose and acesulfame-K are also in accordance with recently published *K*_d_ values measured by the intrinsic fluorescence of hTAS1R2-NTD (40 µM and 100 µM, respectively)^30^. Interestingly, our data revealed a *K*_d_ value of hTAS1R2 for neotame (2.78 µM), is 18-fold lower compared to the *K*_d_ value measured with hTAS1R2-NTD (50 µM). We can speculate that the presence of the TMD may increase the affinity of hTAS1R2 for this sweetener.

Unfortunately, the weak expression of hTAS1R2 on the cell surface does not allow us to obtain sufficient functional activity to determine EC_50_ values using HEK293S-GnTI- cells co-transfected with the plasmid coding for Gα16gust44 and hTAS1R3. To overcome this difficulty, we used HEK293T cells stably transfected with Gα16gust44 and transiently transfected with a plasmid coding for hTAS1R2-FLAG and hTAS1R3-FLAG. We measured a strong expression of each subunit by immunocytochemistry (Supplementary Fig. S5). For functional assay, cells were also transiently transfected with the plasmid pGP-CMV-GCaMP6S to allow production of ultra-sensitive protein calcium sensor^64,65^. Calcium imaging assays led to determination of EC_50_ values in accordance with previously published data^3,12,15,16,26,28,51^. It is interesting to note that even if sucralose, neotame and acesulfame-K were able to bind the VFT of hTAS1R2 subunit^12,15,66^ they were unable to produce biological and functional response of the receptor in absence of hTAS1R3. Sucralose, which interacts with the VFT of hTAS1R3 subunit^15^ is also unable to produce cellular response by itself in absence of hTAS1R2 subunit. This is the same for cyclamate which binds hTAS1R3-TMD and is unable to stimulate transfected cells expressing hTAS1R3 alone. As previously reported, perillartine, which binds hTAS1R2-TMD is able to stimulate cells expressing hTAS1R2 alone and is more effective when both sweet taste receptor subunits are expressed. This difference in response between binding at the receptor level and functional response of the sweet taste receptor at the cellular level could be explained by the mechanism of inter-subunit or intra-subunit rearrangement and the rigidity of the CRD, that lead to conformational changes after ligand binding and finally interaction with G proteins. These arguments are supported by many recent structural studies including one on the Medaka fish taste receptor T1r2-T1r3^14,32^ and other class C GPCR, like CaSR, mGlu and GABA_B_ receptors^45,62,67–70^, which demonstrated that the reorientation of the VFT domain could lead to intra-subunit movement between VTF domain and TMD revealing multiple allosteric interactions and cooperativity between VFT domain, CRD and TMD. This rearrangement could explain why intrinsic tryptophan fluorescence can be measured for perillartine binding in TMD even if only two tryptophan were present in this part of the receptor. On the other hand, it could also suggest that binding sites for perillartine involved TMD1 in addition to TMD3, TMD5 and TMD7 that have been demonstrated with the hTAS1R2-TMD-inhibitor amiloride^28^. At the moment, this is not clear which TMD between TAS1R2 and TAS1R3 could be responsible for coupling to G protein activation. Studies performed on the mGlu2-4 heterodimers show that mGlu4-TMD lead to protein G activation even if mGlu2 homodimer can also do it^62^. Our results on hTAS1R2 homodimer with perillartine suggests that G protein could be activate preferentially by hTAS1R2 subunit, but it has been shown that hTAS1R3 homodimer could also be activated by calcium^71^. As suggested before^72^, we propose that perillartine induces conformation changes in the hTAS1R2-TMD, which in turns leads to inter-subunit rearrangement between the two TMD sufficient to activate hTAS1R2 homodimer and hTAS1R2/hTAS1R3 heterodimers. Inversely, for sucralose, neotame, and acesulfame-K the rearrangement of the VFT domain, or that of cyclamate on the hTAS1R3-TMD, are not sufficient to activate hTAS1R2 or hTAS1R3 homodimers because of difference in energy barrier or because hTAS1R2 play a key role in the activation process. Strikingly, the *K*_d_ values determined for the purified hTAS1R2 receptor were in agreement with the EC_50_ values measured using a cellular assay. Furthermore, these data are in agreement with the relative potencies of sweet tasting compounds (Table 1). Interestingly, we reported for the first time a *K*_d_ value of 373 µM for perillartine, which is able to bind to the TMD of hTAS1R2 and is in accordance with the 61 µM evaluated using a cellular assay on HEK293T-Gα16gust44 transiently transfected with hTAS1R2 alone.

In conclusion, despite its low expression level and weak targeting to the cell surface, we successfully purified functional full-length hTAS1R2 receptor, allowing the performance of biophysical studies and measurement of its affinity for sweet tasting compounds or sweet tasting modulators. The main advantage of the stable expression is the reduction of the transfection costs, including plasmid preparation and transfection reagent, which can be limiting for protein production on a large scale. This approach paves the way also to generate nanobodies for the subsequent analysis of the functional and structural properties of hTAS1R2.

## Materials and methods

The method used for construction of the tetracycline inducible HEK293S stable cell line expressing hTAS1R2, and the following steps of extraction, purification and biophysical characterization were carried out as previously described for the human olfactory receptor hOR1A1^36^ with slight modifications.

### Chemicals

The sweet tasting molecules (sucralose CAS 56038-13-2, neotame CAS 165450-17-9, acesulfame-K CAS 55589-62-3, cyclamate-Na CAS 139-05-9, perillartine CAS 30950-27-7), EZview Red anti-FLAG M2 Affinity Gel, monoclonal ANTI-FLAG M2 antibody and FLAG peptide were purchased from Sigma-Aldrich (Saint-Quentin Fallavier, France). The detergents octyl-β-d-glucoside (OG), n-dodecyl-β-d-maltopyranoside (DDM), fos-choline 14 (FC14) and Lauryl Maltose Neopentyl Glycol (LMNG) were purchased from Anatrace (Affymetrix, High Wycombe, United Kingdom). Stock solutions of sweet tasting molecules (100 mM) were prepared in Dulbecco's phosphate-buffered saline (PBS: 137 mM NaCl, 2.7 mM KCl, 1.8 mM KH_2_PO_4_, 10 mM Na_2_HPO_4_, pH 7.4) with 0.1% detergent for ligand binding titration by fluorimetry. For the cellular assay, sweeteners were freshly prepared and dissolved in buffer C1 (130 mM NaCl, 5 mM KCl, 10 mM HEPES, 2 mM CaCl_2_, 10 mM pyruvic acid, pH 7.4, 300 mOsm). Dulbecco's modified Eagle's medium (DMEM), all tissue culture media components, streptavidin-conjugated Alexa Fluor 568 and goat anti-mouse Alexa 488 secondary antibody were purchased from Life Technologies (St Aubin, France).

### Construction of a tetracycline inducible HEK293S stable cell line expressing hTAS1R2

The sequence encoding hTAS1R2 was obtained from the online UniProtKB Protein database (accession Q8TE23). The gene was synthesized commercially (DNA 2.0, Menlo Park, CA, USA) and optimized for expression in mammalian cells. Protein expression and purification were facilitated by the addition of the Kozak sequence (GCCACCATGG) immediately before the start codon and by the addition of the FLAG epitope tag (DYKDDDDK) to the N-terminus of the hTAS1R2 gene after the starting codon. The synthetic cDNA encoding hTAS1R2 was subcloned into the *Nde*I and *Eco*RI restriction sites of the pcDNA5/TO-inducible expression vector (Invitrogen). The resulting expression vector pcDNA5/TO-hTAS1R2 encodes a fusion protein comprising an N-terminal FLAG-tag followed by hTAS1R2. The plasmid was amplified in *E. coli* DH5α cells and purified with the PureYield Plasmid Midiprep System (Promega, Charbonnières-les-Bains, France).

The pcDNA5/TO-Flag-hTAS1R2 plasmid was transfected into the human inducible N-acetylglucosaminyltransferase I-negative HEK293S cell line (HEK293S GnTI^−^) using Fugene HD (Promega, Madison, Wisconsin, USA)^39^. The HEK293S GnTI^−^ cells were grown in DMEM/F12 supplemented with 10% foetal bovine serum, nonessential amino acids (0.1 mM), 2 mM GlutaMAX (Gibco, Life Technologies), HEPES (15 mM), penicillin (100 units/mL), streptomycin (100 µg/mL) and blasticidin (5 µg/mL) at 37 °C in a humidified atmosphere containing 6.7% CO_2_. The expression and selection of the clones were carried out as previously described^36^ using 125 µg/mL hygromycin. Clones were expanded and screened for the inducible expression of FLAG-tagged hTAS1R2 using media supplemented with 1 µg/mL tetracycline and 5 mM NaBu for 48 h. The expression level of FLAG-hTAS1R2 was estimated by dot blot using mouse anti-FLAG primary antibody (dilution 1/2000). The clone showing the highest level of hTAS1R2 expression under the induction conditions while maintaining undetectable expression without induction was selected and expanded into large-scale culture for use in all subsequent experiments.

### Cell extract preparation

The cell extraction was performed as previously described^36^. Briefly, the hTAS1R2-HEK293S GnTI^−^ cells were grown in T225 flasks for 5 days at 37 °C until they reached 80% confluence. The cells were then incubated in medium containing tetracycline (1 µg/mL) and NaBu (5 mM). After 48 h, the adherent cells and cells in suspension were harvested into ice-cold medium, pelleted by centrifugation at 800*g* for 15 min at 4 °C and washed with PBS containing a protease inhibitor cocktail (Sigma-Aldrich). Cells pellets from 20 flasks were then pooled and centrifuged again. The pooled pellet was flash frozen in liquid nitrogen and stored at − 80 °C until purification.

On the day of purification, the cell pellet was thawed on wet ice. The FLAG-tagged hTAS1R2 protein was solubilized by resuspending the cell pellet in ice-cold PBS buffer containing 2% w/v LMNG and a protease inhibitor cocktail (2 mL per T175 flask). The cell homogenate was sonicated for 1 min using a Vibracell 750 sonicator (Sonics & Materials, Newtown, USA) equipped with a 2 mm tip and set to 30% maximum power. The homogenate was further disrupted by high-speed shaking with a tissue lyser (TissueLyser, Qiagen, Hilden, Germany) for 3 min after carbon beads were introduced into each microtube. Finally, the homogenate was incubated for 2 h at 4 °C under strong agitation using a Vortex Genie II mixer (Scientific Industries, Bohemia, USA) and then centrifuged at 105,000*g* for 1 h at 4 °C. The resulting supernatant was immediately submitted to immunoaffinity purification.

### hTAS1R2 purification by immunoaffinity chromatography

To purify the FLAG-tagged hTAS1R2 protein from the cell extract, we followed the protocol already described^36^ using the EZview Red anti-FLAG^®^ M2 affinity gel, in which the monoclonal antibody ANTI-FLAG M2 is covalently attached to cross-linked agarose beads. The cell homogenate was mixed with the EZview Red anti-FLAG M2 beads (binding capacity: 0.6 mg/mL) and rotated for 2 h at 4 °C to capture the FLAG-tagged hTAS1R2. The beads were then transferred into Pierce spin columns and collected by centrifugation at 1500*g* for 1 min. Then, the beads were washed 5 times with cold PBS containing 0.1% LMNG. After the last wash, the FLAG-tagged hTAS1R2 bound to the beads was eluted by competitive elution with 5 column volumes of a PBS-0.1% LMNG solution containing 100 mg/mL FLAG peptide. The eluate was loaded on SDS-PAGE, stained by Coomassie blue and analysed by western blot.

### Size exclusion chromatography (SEC)

The FLAG-tagged hTAS1R2 samples that had been eluted from the ANTI-FLAG M2 beads were pooled and concentrated to 0.3–0.5 mg/mL using a 30-kDa MWCO filter column (Vivaspin, Sartorius, Aubagne, France). Then the concentrated FLAG-tagged hTAS1R2 was purified by gel filtration as previously reported^36^. The samples were then loaded for gel filtration chromatography (Superdex 200 Increase 10/300GL column) on an Äkta Pure fast protein liquid chromatography system (GE Healthcare, Velizy-Villacoublay, France). The column was equilibrated with 2 column volumes of wash buffer (PBS, 0.1% LMNG, pH 7.3) before the immunopurified FLAG-tagged hTAS1R2 sample was applied. After loading, the column was rinsed with wash buffer at 0.5 mL/min, and the column flow through was monitored by UV absorbance at 280 nm. The molecular masses of the FLAG-tagged hTAS1R2-detergent complexes were estimated by calibrating the column with a gel filtration standard mixture (Sigma-Aldrich). The following standard proteins were used: thyroglobulin (669 kDa), β-amylase (200 kDa), alcohol dehydrogenase (150 kDa), monomeric BSA (66 kDa), carbonic anhydrase (29 kDa), myoglobin (17 kDa) and lysozyme (14.3 kDa). The protein fractions (0.5 mL) were collected using an automated fraction collector. The collected fractions were deposited on SDS-PAGE, stained by Coomassie blue and subjected to immunoblotting analysis.

### SEC coupled with multi-angle light scattering (SEC-MALS) analysis

The oligomeric state of the purified FLAG-tagged hTAS1R2 protein was determined using an SEC column coupled to a MALS detector. SEC was performed using a Superdex 200 Increase 3.2/300 column (GE Healthcare) and eluted with PBS containing 0.1% LMNG (pH 7.3) at 0.1 mL/min. The protein was detected with a triple-angle light scattering detector (Mini-DAWN TREOS, Wyatt Technology) connected to a UV detector (UV 100 SpectraSeries, Thermo Separation Products, Waltham, MA, USA) operating at a wavelength of 280 nm and a differential refractometer (RiD-10A, Shimadzu, Kyoto, Japan). A 100 µL volume of each sample was injected onto the column. The molecular weights of the protein detergent complexes were determined with ASTRA VI software (Wyatt Technology Santa Barbara, CA, USA). A specific refractive index increment (dn/dc value), which was estimated at 0.185 mL/g, was used for mass calculation. The suitability of the system was assessed by analysing the standard proteins BSA (66 kDa) and β-amylase (200 kDa).

### hTAS1R2 secondary structure analysis using circular dichroism spectroscopy

The circular dichroism (CD) spectra of the FLAG-tagged hTAS1R2 samples were recorded at 20 °C using a J-815 Jasco spectropolarimeter (Jasco, Tokyo, Japan) equipped with a Peltier temperature control. The CD spectra were corrected for the buffer and ligand contributions and converted to mean residue ellipticity in deg cm^2^ dmol^−1^. The spectra recorded between 178 and 260 nm were averaged over 5 scans accumulated at 0.5 nm intervals with a 50 nm/min scan speed and 5 s of response time. Spectra were smoothed using the Savitzky-Golay convolution filter with a span of 15. The secondary structure proportions were estimated using the deconvolution CDSSTR algorithm available on the DichroWeb website (http://dichroweb.cryst.bbk.ac.uk/html/home.shtml)^73^.

### Intrinsic tryptophan fluorescence measurements

Intrinsic fluorescence spectra were recorded with a Cary Eclipse spectrofluorimeter (Varian Instruments, Les Ulis, France) equipped with a Peltier temperature control unit. The temperature was maintained at 20 °C. FLAG-tagged hTAS1R2 protein (0.10 µM in PBS, 0.1% LMNG buffer) was excited at 280 nm, and the emission spectra were recorded from 300 to 400 nm, with a 10 nm bandwidth for both excitation and emission in the presence and absence of ligands. The sweetener solutions were freshly prepared in PBS-0.1% LMNG buffer. Successive aliquots of ligand solutions were added to 400 µL of the FLAG-tagged hTAS1R2 solution. A range of ligand concentrations adapted to each compound was tested (0.1 µM to 630 µM for sucralose, 0.1 nM to 100 µM for neotame, 0.1 to 2400 µM for acesulfame-K, 0.1 µM to 1 mM for perillartine and 0.1 µM to 10 mM for cyclamate). The fluorescence measurements were corrected for bleaching and nonspecific buffer quenching. The dissociation constants (*K*_d_) were calculated from a plot of the fluorescence intensity measured at the maximum emission wavelength (340 nm) versus the concentration of total ligand obtained with a standard nonlinear regression method using SigmaPlot software (Systat Software Inc., San Jose, CA, USA). The reported *K*_d_ values are the average of triplicate measurements performed on at least three independently purified protein samples.

### Immunoblotting analysis of hTAS1R2

The fraction eluted from gel filtration was concentrated by methanol precipitation at − 20 °C for 1 h, followed by 12,000*g* centrifugation at 4 °C for 1 h. Pellets were resuspended in 100 µL of buffer (4% SDS, 0.004% bromophenol blue, 0.125 M Tris–HCl pH 6.8, 20% glycerol, 10% β-mercapto ethanol). The samples (50 µL/well) were loaded onto 4–15% SDS-PAGE. The molecular weight markers (Dual Xtra Standards, Bio-Rad) were loaded in the first lane of the gel. SDS-PAGE was performed using a Mini-Protean II system (Bio-Rad). Following electrophoresis at 200 V for 40 min, the proteins were transferred to polyvinylidene fluoride membranes (Trans-lot Turbo PVDF transfer pack, Bio-Rad) using a Trans-Blot Turbo transfer system from Bio-Rad at 1.3 A, 25 V for 10 min. The membranes were blocked in a solution containing 10 mM Tris–HCl pH 8.0, 150 mM NaCl, 0.05% Tween 20 and 5% non-fat dry milk (TBS-T) for 1 h at room temperature. The blots were then incubated with the mouse anti-FLAG M2 primary antibody, diluted at 1/2000 in TBS-T, for 1 h at 4 °C. After washing (five 5 min washes with TBS-T), the membranes were incubated with goat anti-mouse horseradish peroxidase conjugated secondary antibody (diluted 1:25,000) for 1 h at room temperature and then rinsed five times with TBS-T. The protein antibody complexes were detected using an ECL chemiluminescence kit (Clarity Western ECL Substrate, Bio-Rad) and the ChemiDoc MP Imaging System (Bio-Rad).

### Immunocytochemistry

For immunocytochemical staining analyses, we performed the protocol as reported earlier^36^. The stable hTAS1R2-HEK293S GnTI^−^ clones were seeded on 4-well culture slides (Corning Inc., Corning, NY, USA) precoated with BD Cell-Tak adhesive (Corning). When the cells reached ~ 90% confluence, they were treated with 1 µg/mL tetracycline and 5 mM NaBu for 48 h. Then, the cells were rinsed twice with Hank's HEPES-balanced salt solution and permeabilized for 5 min in cold acetone-methanol (1:1). To visualize the FLAG-tagged hTAS1R2 expression level, the cells were blocked with 5% goat serum in PBS for 30 min at 25 °C and incubated for 1 h at 25 °C with a 1/500 dilution of the mouse anti-FLAG M2 primary antibody in an antibody diluent (Dako Les Ulis, France). The cells were then rinsed twice with PBS for 5 min and incubated with a 1/400 dilution of the Alexa Fluor 488-conjugated goat anti-mouse IgG secondary antibody (Life Technologies) in the Dako antibody diluent for 1 h at 25 °C to visualize FLAG-tagged hTAS1R2. The cells were analysed using an epi-fluorescence inverted microscope (Eclipse TiE, Nikon, Champigny sur Marne, France) equipped with an 20× objective lens and a LucaR EMCCD camera (Andor Technology, Belfast, UK). To study the subcellular localization of FLAG-tagged hTAS1R2, the receptor was analysed as previously described. In addition, to visualize the plasma membrane, the cells were cooled on ice for 30 min and then incubated with 2 mg/mL biotin-concanavalin A for 1 h on ice before permeabilization. The plasma membrane was then labelled with Alexa Fluor 568 streptavidin conjugate (dilution 1/500; Life Technologies). After washing the cells with PBS, the chambers were detached from the slide and the coverslips were placed with mounting medium (Dako). The cells were analysed using a two-photon confocal microscope (Nikon A1-MP) equipped with an 60× objective lens (DImaCell platform, University of Burgundy, Dijon, France).

### Heterologous expression and calcium assay

We used a calcium imaging assay to establish dose–response curves for the sweet taste receptor hTAS1R2/hTAS1R3 and determine EC_50_ values for the sweet stimuli previously tested in spectrofluorimetric experiments. We cloned the cDNAs coding hTAS1R2 and hTAS1R3 subunits into pcDNA6 and pcDNA4 expression plasmids, respectively, and we added FLAG tag to C-terminus of each construct to measure protein expression level by immunocytochemistry as described previously. HEK293T cells stably transfected with Gα16gust44 were seeded at a density of 0.35 × 10^6^ cells per well into 96-well black walled, clear bottom microtiter plates (Falcon) in high-glucose DMEM supplemented with 2 mM GlutaMAX, 10% dialyzed foetal bovine serum, penicillin/streptomycin and G418 (400 µg/mL) at 37 °C and 6.3% CO_2_, in a humidified atmosphere. After 48 h, the cells were transiently transfected with each TAS1R subunit (60 ng/well for each plasmid) and plasmid pGP-CMV-GCaMP6S (Addgene #40753, 50 ng per well) coding for a green fluorescent protein-based calcium indicator, using Fugene HD (0.4 µL/well). After a further 24 h incubation, the cells were washed twice with buffer C1 and then stimulated with sweet tasting compounds. After excitation at 488 nm, calcium responses were recorded at 510 nm using a Molecular Devices FlexStation 3 system. Acquisition was made simultaneously from 8 wells corresponding to the range of taste solutions. We averaged the responses of 18 wells receiving the same stimulus one three independent experiments. We subtracted the mean calcium traces of mock-transfected cells stimulated with the same concentration of stimulus. Plots of the fluorescence variation amplitude versus concentration were fitted by four-parameter logistic nonlinear regression allowing us to calculate the EC_50_ values of activation.

## Supplementary Information

Supplementary Figures.
