# Supplementary material for: Biophysical and functional characterization of the human TAS1R2 sweet taste receptor overexpressed in a HEK293S inducible cell line
Source: Sci Rep. 2021 Nov 15;11:22238. doi: 10.1038/s41598-021-01731-3 (PMC8593021; doi:10.1038/s41598-021-01731-3)
Supplement: Supplementary file 1 — Supplementary Figures. [file 41598_2021_1731_MOESM1_ESM.docx]

**Biophysical and functional characterization of the human TAS1R2 sweet taste receptor overexpressed in a HEK293S inducible cell line**

**Christine Belloir**^1^**, Marine Brulé**^1^**, Lucie Tornier**^1^**, Fabrice Neiers**^1^**, and Loïc Briand**^1, *^

^1^ Centre des Sciences du Goût et de l'Alimentation, AgroSup Dijon, CNRS, INRAE, Université Bourgogne Franche-Comté, F-21000, France

* loic.briand@inrae.fr

**Supplementary data**

**
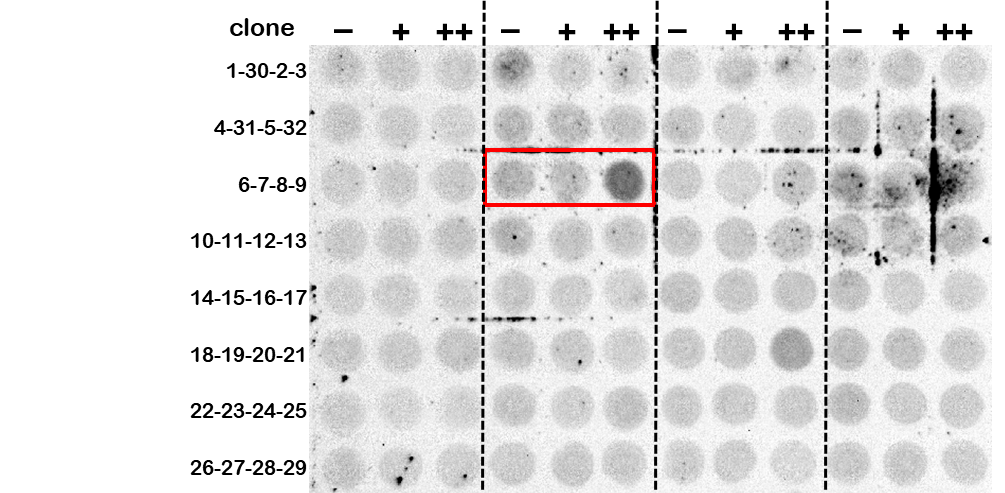
**

**Figure S1. Dot blot analyses of HEK293S GnTI- cell clones that were stably transfected with the pcDNA5/TO-FLAG-hTAS1R2 plasmid**. The cell clones were treated with plain media (-) or media supplemented with 1 mg/mL tetracycline (+) or tetracycline plus 5mM NaBu (++) for 48H. The levels of hTAS1R2 in the cell lysates were probed using the anti-FLAG M2 antibody (3 µL drop containing 3 µg of protein were deposited for each analysis). Clone 7 (in red box) showed the highest level of induction under tetracycline and NaBu and, therefore was selected for all subsequent experiments.

**
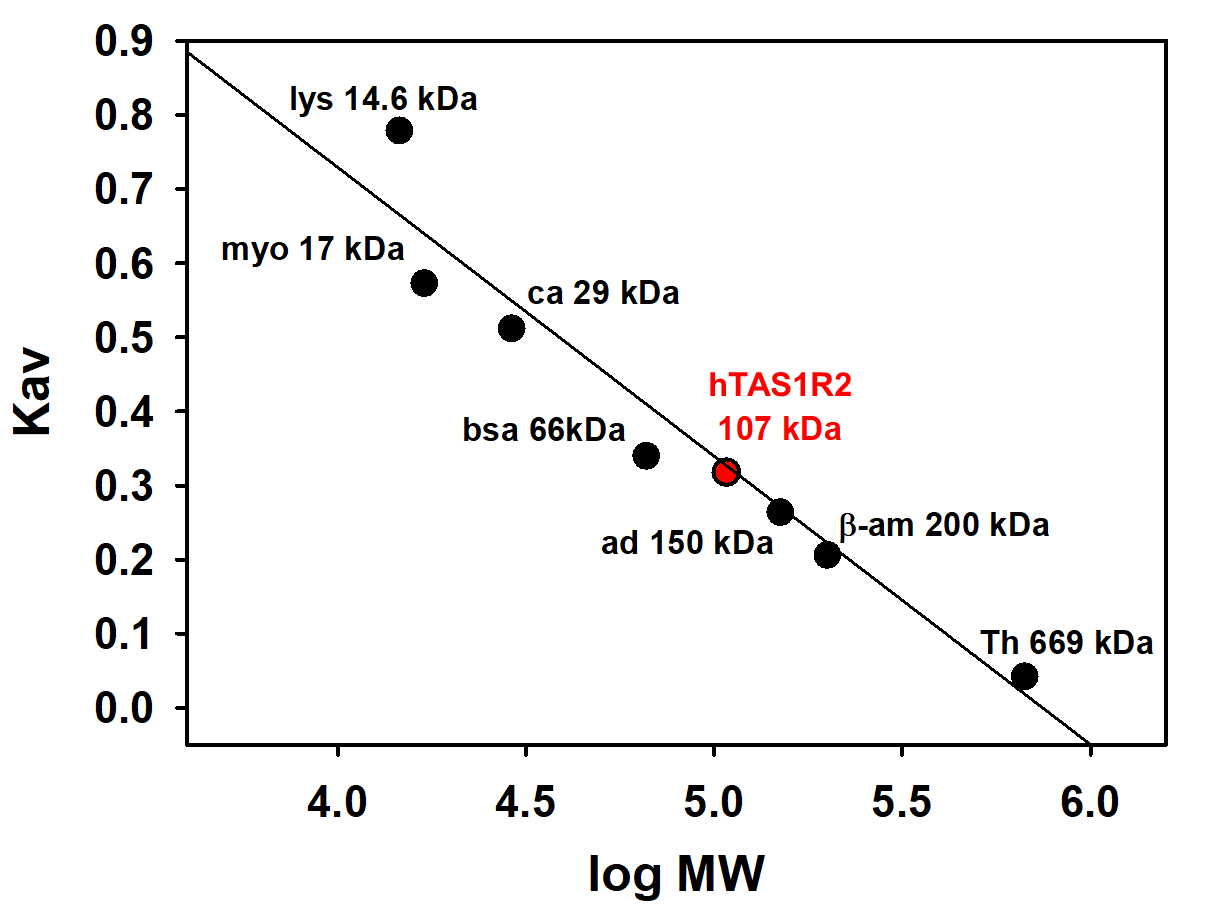
**

**Figure S2. Gel filtration chromatography of the purified hTAS1R2.** The calibration curve for the Superdex 200 Increase 10/300GL column (GE Healthcare) was established with thyroglobulin (Th, 669 kDa) β-amylase (β-am, 200 kDa), alcohol dehydrogenase (ad, 150 kDa), monomeric BSA (bsa, 66 kDa), carbonic anhydrase (ca, 29 kDa), myoglobin (myo,17 kDa) and lysozyme (lys,14.6 kDa). The estimated molecular mass of hTAS1R2 is 107 kDa.


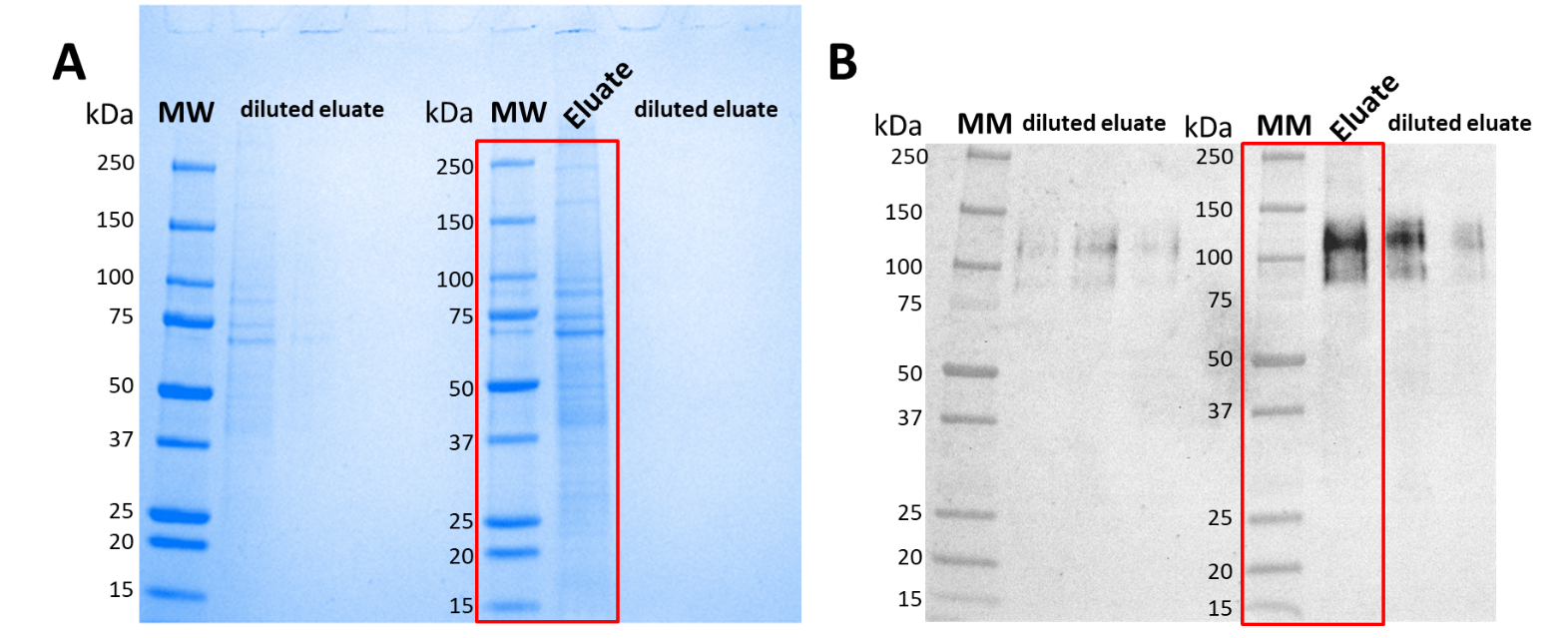


**Figure S3. Analysis of immunoaffinity purified hTAS1R2.** FLAG-tagged hTAS1R2 was solubilized in PBS containing 2% LMNG and captured using the EZview Red anti-FLAG M2 affinity gel. After elution with FLAG peptide, the eluate was collected, concentrated and subjected to SDS-PAGE followed by (A) staining with Coomassie blue and (B) by western blotting using mouse anti-FLAG M2 primary antibody and goat anti-mouse horseradish peroxidase conjugated secondary antibody. The red boxes on these original SDS-PAGE and western blot denoted the regions cropped for the Figure 4 shown in the manuscript.


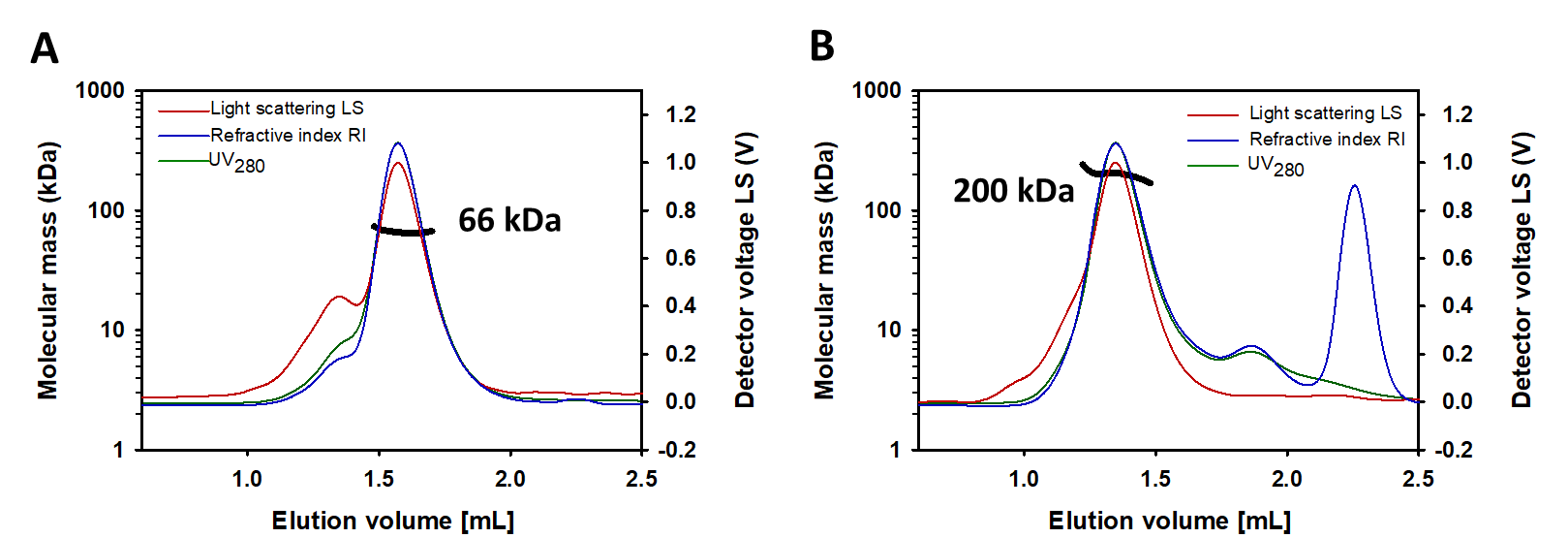


**Figure S4. Standard calibration of SEC-MALS.** A standard solution of 1 mg/mL (A) serum albumin bovine serum albumin (BSA) or (B) β-amylase prepared in the gel filtration buffer (PBS with 0.1% LMNG) was injected on a gel filtration Superdex 200 3.2/300 column (GE Healthcare) to calibrate the SEC-MALS analysis. The chromatograms show the readings of the light scattering (LS), the differential refractive index (dRI) and UV detectors in red, blue and green, respectively. The scale for the LS detector is shown on the right-hand axis. The thick black line indicates the calculated molecular mass of the eluting protein throughout the chromatogram (scale on the left-hand axis). BSA has a fitted molecular mass of 66 kDa in monomeric form and β-amylase has a fitted molecular mass of 200 kDa, which is in accordance with their theoretical monomer molecular mass values of 66.5 kDa and 200 kDa, respectively.


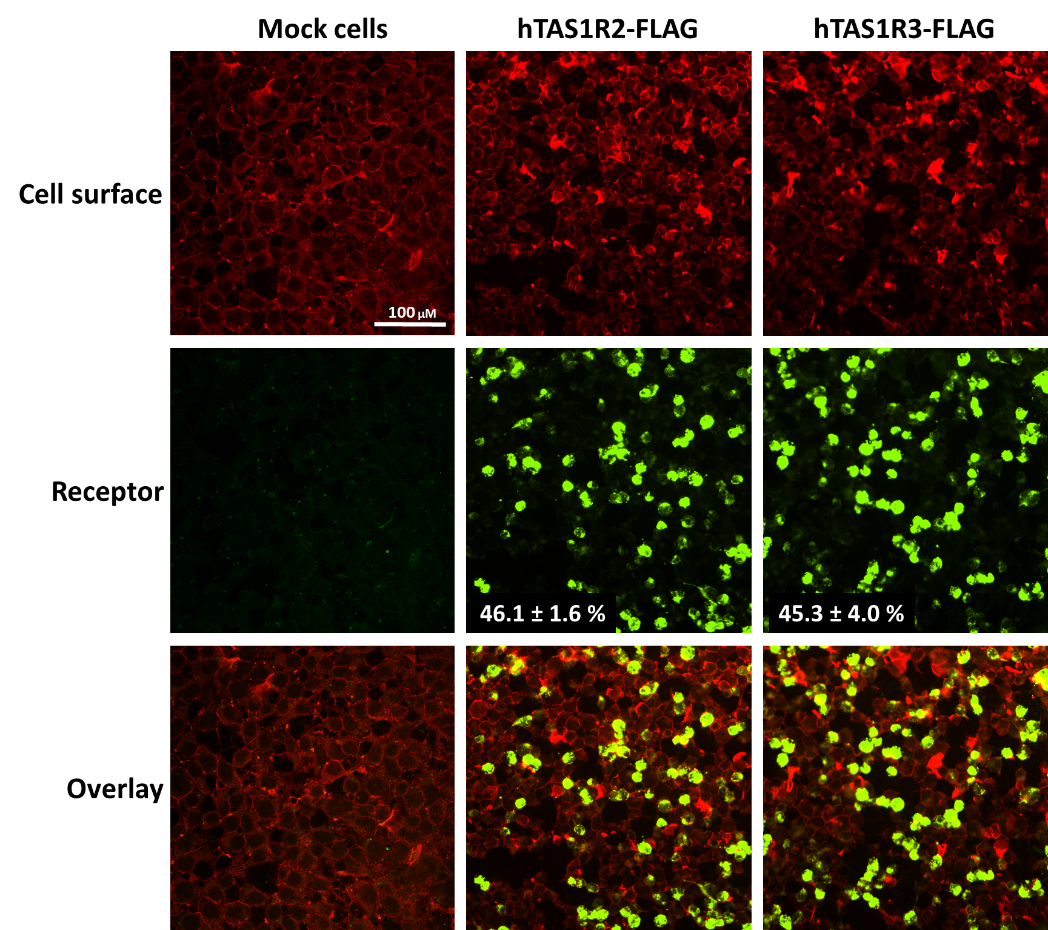


**Figure S5. Immunocytochemistry of HEK293T Gα16gust 44 expressing hTAS1R2-FLAG or hTAS1R3-FLAG.** Cells were transiently transfected with pcDNA6-hTAS1R2-FLAG + pcDNA4-hTAS1R3 or with pcDNA4-hTAS1R3-FLAG + pcDNA6-hTAS1R2 using Fugene HD. The expression level of hTAS1R2-FLAG receptor or hTAS1R3-FLAG receptor (shown in green) were detected using a primary anti-FLAG M2 antibody and a fluorescently labelled secondary antibody (Alexa Fluor 488). The cell surface (shown in red) was detected by biotin-conjugated concanavalin A and streptavidin-conjugated Alexa Fluor 568. The cells were analysed using an epi-fluorescence inverted microscope (Eclipse TiE, Nikon, Champigny sur Marne, France) equipped with an x20 objective lens and a LucaR EMCCD camera (Andor Technology, Belfast, UK). The percentage of cells expressing hTAS1R subunit was measured as mean ± sem of six images.
